# CircHIPK3 regulates cardiac fibroblast proliferation, migration and phenotypic switching through the miR-152-3p/TGF-β2 axis under hypoxia

**DOI:** 10.7717/peerj.9796

**Published:** 2020-08-25

**Authors:** Weiwei Liu, Yan Wang, Zhimei Qiu, Ranzun Zhao, Zhijiang Liu, Wenming Chen, Junbo Ge, Bei Shi

**Affiliations:** 1 Department of Cardiology, Affiliated Hospital of Zunyi Medical University, Zunyi, China; 2 Department of Cardiology, Shanghai Institute of Cardiovascular Diseases, Zhongshan Hospital, Fudan University, Shanghai, China

**Keywords:** CircHIPK3, MiR-152-3p, TGF-β2, Myofibroblast transition, Hypoxia, Cardiac fibroblast

## Abstract

**Background:**

The occurrence of pathological cardiac fibrosis is attributed to tissue hypoxia. Circular RNAs play significant regulatory roles in multiple cardiovascular diseases and are involved in the regulation of physiological and pathophysiological processes. CircHIPK3 has been identified as the one of the most crucial regulators in cardiac fibrosis. However, the mechanisms by which circHIPK3 regulates cardiac fibrosis under hypoxia remain unclear. Our study aimed to determine circHIPK3 expression in cardiac fibroblasts (CFs) and investigate the functions of circHIPK3 in hypoxia environment.

**Methods:**

The expression level of circHIPK3 in CFs under hypoxia (1% O_2_) was analyzed by qRT-PCR. The role of circHIPK3 on the proliferation and migration of CFs were determined by EdU, cell wound scratch assay and cell cycle. The expression of proteins associated with phenotypic transformation in CFs in vitro was examined by immunofluorescence assay and western blot. Bioinformatics analysis, dual luciferase activity assay and RNA fluorescent in situ hybridization assay revealed that miR-152-3p was identified as a target of circHIPK3 and that TGF-β2 was targeted by miR-152-3p.

**Results:**

CircHIPK3 expression was significantly upregulated in CFs in a hypoxic environment. In vitro, overexpressing circHIPK3 obviously promoted CF proliferation, migration and phenotypic changes under hypoxia, but those processes were suppressed by circHIPK3 silencing. CircHIPK3 acted as an endogenous miR-152-3p sponge and miR-152-3p aggravated circHIPK3 silencing induced inhibition of CF proliferation, migration, phenotypic transformation and TGF-β2 expression in vitro. In summary, circHIPK3 plays a pivotal role in the development of cardiac fibrosis by targeting the miR-152-3p/TGF-β2 axis.

**Conclusions:**

These findings demonstrated that circHIPK3 acted as a miR-152-3p sponge to regulate CF proliferation, migration and phenotypic transformation through TGF-β2, revealing that modulation of circHIPK3 expression may represent a potential target to promote the transition of hypoxia-induced CFs to myofibroblasts.

## Introduction

Ischemic heart disease is a leading cause of morbidity and mortality around the world (Sivanesan et al., 2018). Cardiac ischemic injury can induce a remodeling response that involves fibroblast activation (Lacraz et al., 2017). The activation of fibroblasts, which induces the synthesis and accumulation of extracellular matrix (ECM) proteins, such as collagen types I and III, and promotes the expression of α-smooth muscle actin (α-SMA) (Maruyama et al., 2018; Russo et al., 2019; Wang et al., 2014), causes myocardial stiffening and remodeling to support the structure of the heart. Cardiac fibroblasts (CFs) play a vital role in the repair and remodeling response and have the ability to resist hypoxia after ischemic injury (Li, Dietz & Von Harsdorf, 1999; Woodall et al., 2016). It has been reported that hypoxia is involved in the development of cardiac fibrosis (Bao et al., 2020; Kumar et al., 2019; Watson et al., 2014). Moreover, hypoxia can induce cardiac fibroblast proliferation and phenotypic transformation (Gao et al., 2014). However, the mechanism of cardiac fibrosis involved in CFs exposed to hypoxia remains to be revealed.

Circular RNAs (circRNAs) belong to a large type of endogenous RNAs molecule that characterized by a covalent ring structure without 5′ caps and 3′ tails (Yu & Kuo, 2019). Emerging evidence has shown that circRNAs are involved in cardiac fibrosis progression (Gu et al., 2020; Zhang et al., 2019b). Moreover, circRNAs could specifically function as microRNA (miRNA) sponges, which serve as an endogenous regulatory mechanism to sequester sequence-specific miRNAs and regulate miRNAs distribution on their mRNA targets (Tay et al., 2015), to regulate alternative splicing, and modulate the expression of target genes. For instance, circRNA_010567 is reported to sponge miR-141 to inhibit the expression of TGF-β1 and promote cardiac fibrosis (Zhou & Yu, 2017). Our previous findings showed that hypoxic preconditioning enhanced the expression of circHIPK3 in exosomes from cardiomyocytes. Exosomal circHIPK3 protected cardiac microvascular endothelial cells from oxidative damage through the miR-29a/IGF-1 signaling pathway (Wang et al., 2019b). CircHIPK3, a particularly widespread and abundant circRNA, has been verified to be involved in regulating the developmental and physiological processes of diseases, including survival, migration, and angiogenesis, by sponging different miRNAs (Shan et al., 2017; Wei et al., 2020; Zheng et al., 2016). However, little is known about the function and mechanism of circHIPK3 in cardiac fibrosis under hypoxic conditions. To address this question, we used reverse transcription-quantitative PCR (RT-qPCR) to detect the expression of circHIPK3, and the results showed that circHIPK3 was upregulated in vitro in CFs exposed to hypoxia. CircHIPK3 silencing suppressed CF proliferation, migration and the development of cardiac fibrosis. Moreover, miR-152-3p overexpression reinforced the inhibitory effect of circHIPK3 silencing in CFs. Our results have revealed a novel mechanism for the development of cardiac fibrosis in which circHIPK3 acts as a miR-152-3p sponge to promote TGF-β2 expression and regulate cardiac fibroblast transformation into myofibroblasts.

## Materials and Methods

### Animals

C57BL/6J neonatal mice (1–3 days old) were obtained and housed at Zunyi Medical University (Zunyi, China) and maintained under SPF conditions. All mice were lived with their mothers and were breastfed. The mice were exposed to light for 12 h a day. All experimental animals were used for the isolation of cardiac fibroblast. According to the review of animal experiment Ethics Committee of Zunyi Medical University (Approval No. ZMUER2018-2-177), the experimental scheme followed the animal welfare and ethical principles and met the requirements of ethical norms.

### Culture and treatment of cardiac fibroblasts

Primary CFs were isolated and cultured according to the previously described and slightly improved (Chowdhury et al., 2017). Ventricles were obtained from 1 to 3 days old C57BL/6J neonatal mice. CFs were isolated by enzymatic dissociation methods using 0.08% trypsin and 0.1% collagenase type II (Sigma–Aldrich, Shanghai, China) in *D*-Hank’s balanced salt solution at a 37 °C water bath for 1 h. The cells then were filtered with a 200 mesh screen. Cells were cultured in Dulbecco’s modified Eagle’s medium (Gibco, Dublin, Ireland) supplemented with L-glutamine, fetal bovine serum and penicillin-streptomycin. After 1.5 h, CFs were obtained by removing the cell suspension, and then fresh medium was added for further culture.

Cardiac fibroblasts were subjected to hypoxia. The logarithmic growth cells were incubated in a 94% N_2_, 5% CO_2_, and 1% O_2_ gas mixture incubator (Thermo, Waltham, MA, USA) at 37 °C for 6, 12, 24, and 48 h. Cells viability was assessed by Cell Counting Kit-8 (CCK-8) assay.

### Reverse transcription-quantitative PCR

Total cellular RNA was isolated using TRIzol reagent (Life Technologies, Carlsbad, CA, USA). For circRNA isolation, 4U RNase R (Geneseed, Guangzhou, China) was added to one μg total RNA and incubated at 37 °C for 30 min. cDNA synthesis and RT-qPCR analysis were implemented using PrimeScript RT Master Mix and TB Green Premix Ex Taq II kit (Takara, Dalian, China). To quantify the relative expression levels of miRNA, total RNA was reversed to first-strand cDNA with miRNA 1st Strand cDNA Synthesis Kit (Sangon Biotech, Shanghai, China). GAPDH and U6 primers were used as normalization control for circRNA and miRNA expression, respectively. Relative quantification of mRNA expression after normalization of the transcript amount to the endogenous control was calculated using the 2^−ΔΔCt^ method (Tabatabaeian & Hojati, 2013).

### 5-Ethynyl-2′-deoxyuridine incorporation assay

Cell proliferation was analyzed with Cell-Light 5-Ethynyl-2′-deoxyuridine (EdU) DNA Cell Proliferation Kit (RiboBio, Guangzhou, China) according to previously reported (Shan et al., 2017). Briefly, approximately 5 × 10^6^/ml logarithmic growth cells were cultured in confocal dish and placed in an incubator. CFs were labeled with 10 mM EdU working solution for 4 h. Subsequently, the working solution was discarded, and added 4% paraformaldehyde to fix the cells at room temperature. Apollo dye solution was labeled proliferating cells for 30 min in the dark, and then Hoechst 33342 solution was stained cell nuclei. Images were captured by fluorescence microscopy (Olympus, Tokyo, Japan).

### Cell cycle assay

Assessment of cell proliferation was carried out using a Cell Cycle Analysis Kit (4A Biotech, Beijing, China) by FCM. Briefly, CFs were fixed with 95% anhydrous ethanol and kept at 4 °C before DNA staining with PI/RNaseA dye solution for 30 min away from light at 37 °C. Samples were acquired with a flow cytometer (BD Biosciences, San Jose, CA, USA).

### Cell wound scratch assay

Cell migration was assessed using traditional cell wound scratch assay. Briefly, CFs were seeded in six-well plates. A cell-free zone was gently formed on a cell monolayer with a 200 μl plastic pipette tip when the cells reached ~80% confluence, then cells were washed with PBS to remove scraped cell debris. Images were acquired by microscopy. Cell migration was determined by the width of the gaps; the shorter the gaps, the higher the migration capacity.

### Western blot analysis

Western blot analysis was performed as described by us (Wang et al., 2019b). Briefly, proteins was extracted from CFs with RIPA lysis buffer (Biosharp, Hefie, China) and quantified with a BCA protein assay reagent (Beyotime, Haimen, China). Equal of protein lysates was loaded into per lane for SDS-PAGE detection before transferred to PVDF membranes (Millipore, Billerica, MA, USA). Next, all membranes were blocked with 5% nonfat milk solution in TBST buffer for 1 h at room temperature and then incubated with primary antibodies on a shaker at 4 °C overnight. The primary anti-bodies: Anti-Collagen I (Cell Signaling Technology, 91144, dilution: 1:1,000), Anti-Collagen III (Abcam, ab7778, dilution: 1:7,000), Anti-α-SMA (Cell Signaling Technology, 19245, dilution: 1:1,000), Anti-p-Smad2 (Cell Signaling Technology, 18338, dilution: 1:1,000), Anti-p-Smad3 (Cell Signaling Technology, 9520, dilution: 1:1,000), Anti-TGF-β2 (Bioss, bs-20412R, dilution: 1:1,000) and Anti-β-actin Antibody (Abcam, ab8227, dilution: 1:1,000), were used. The next day, membranes were incubated with secondary antibody (Proteintech, SA00001-2, dilution: 1:5,000) for 1 h at room temperature. And then the membranes were detected with ECL chemiluminescent substrate (Biosharp, Hefie, China). β-actin acted as the internal standard.

### Cell transfection

Cardiac fibroblasts were transfected with lentiviral constructs (HanBio, Shanghai, China). SiRNAs were provided by Sigma–Aldrich. In addition, transfection of miR-152-3p mimic or miR-152-3p inhibitor with the riboFECT CP Transfection Kit (RiboBio, Guangzhou, China) was performed. The efficiency and function assay were confirmed 48 h after transfection. Transfection was conducted according to the reagent instructions.

### RNA fluorescent in situ hybridization assay

RNA fluorescent in situ hybridization (RNA FISH) assay was performed as described previously (Shan et al., 2017). Cy3-labeled circHIPK3 and FITC-labeled miR-152 probes were transfected into CFs in six-well plates. The fluorescent signals were detected with a Fluorescent in Situ Hybridization Kit according to the reagent guidelines. Cell nuclei were counterstained with DAPI. The images were acquired with a fluorescence microscope.

### Dual luciferase reporter assay

Firstly, the pmirGLO luciferase reporter plasmid, which contained the potential circHIPK3 and TGF-β2 binding site sequences in miR-152-3p, were constructed (GeneCreate, Wuhan, China). HEK-293T cells were cotransfected with wild-type or mutated pmirGLO-circHIPK3 reporter plasmid and miR-152-3p mimics or negative control (NC). Similarly, the pmirGLO-TGF-β2 reporter vector was cotransfected with miR-152. Firefly luciferase activity was standardized to Renilla luciferase activity.

### Statistical analysis

SPSS 20.0 and GraphPad Prism 8.0 software were used to assess data and for statistical analysis. Kolmogorov–Smirnov test detected that the data were normally distributed. The Student *t* test was used to analyze the differences between two groups, one-way analysis of variance (ANOVA) was suitable for multiple groups. After ANOVA analyses, LDS method was used for the homogeneity of variance，Tamhane’s T2 method was used for variances. The difference was statistically significant with *P*-value < 0.05.

## Results

### CircHIPK3 is upregulated in hypoxia-induced cardiac fibroblasts, which affects cell proliferation, migration, and phenotypic modulation in vitro

CircBase retrieval revealed that the Hipk3 host gene might produce three circRNAs, named circHIPK3, in the mouse genome. One of them, circHIPK3 (mmu_circ_0001052), was highly conserved. Our previous research illustrated that circHIPK3 (mmu_circ_0001052) was upregulated in exosomes released from cardiomyocytes that had been exposed to hypoxia (Wang et al., 2019b). Moreover, circHIPK3 has been reported to be enriched in human fibroblasts (Jeck et al., 2013). This urged us to further investigate the function of circHIPK3 in cardiac fibrosis in CFs under hypoxia.

**Figure 1:**
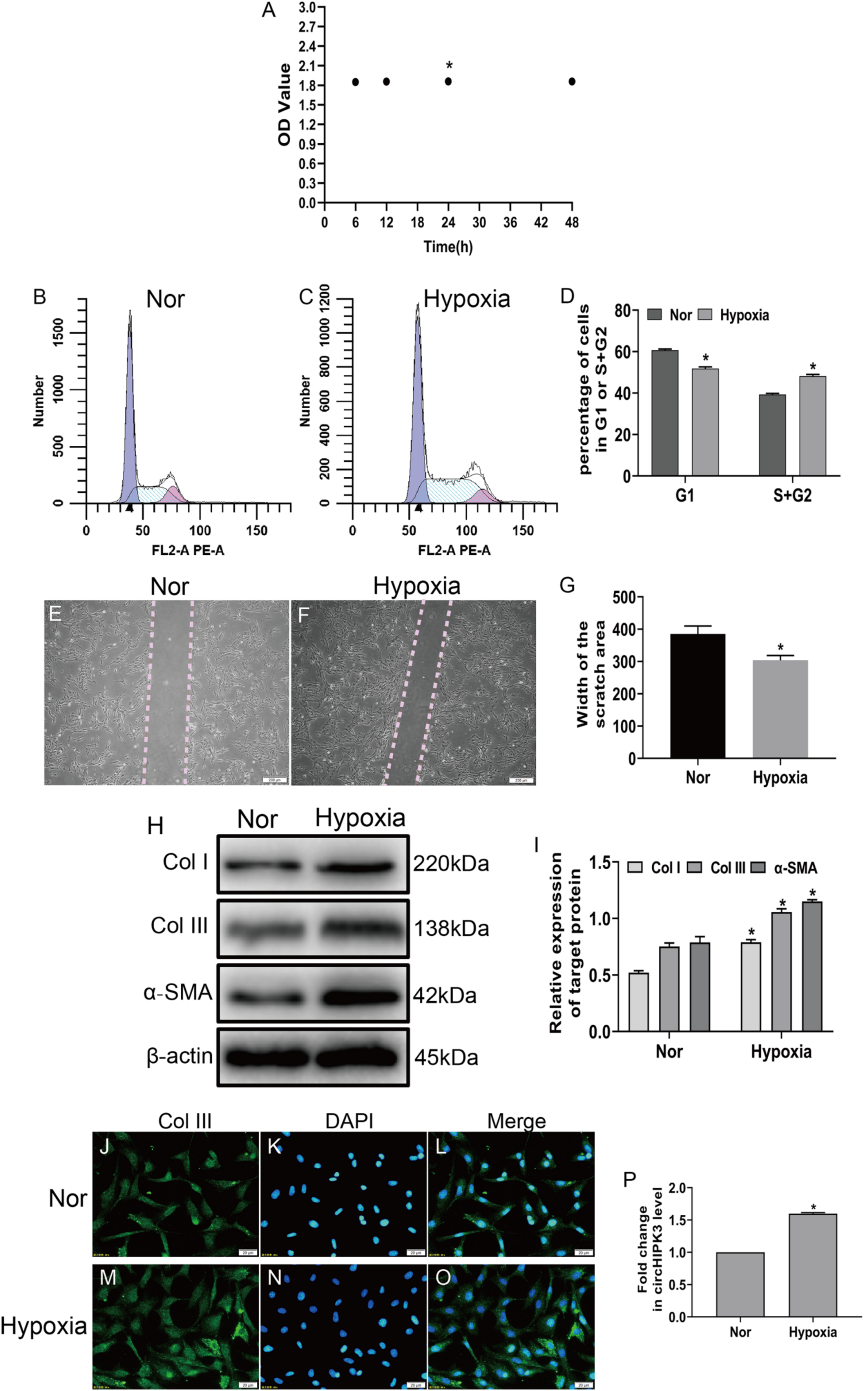
circHIPK3 is upregulated in cardiac fibroblasts and accelerates the process of fibrosis. (A) The OD values from the CCK-8 analysis revealed cell viability of cardiac fibroblasts treated with hypoxia at different times. *n* = 4. *P < 0.05. (B–D) The cell cycle was assayed by a cell cycle assay in cardiac fibroblasts. The results represent the percentage of cells in G1 and S + G2/M phases for the indicated condition. *n* = 3. *P < 0.05. (E–G) Wound scratch assay for the normal (Nor) and hypoxia groups. The average sizes of the gaps were measured at 48 h. Scale bar = 200 μm. *n* = 5. *P < 0.05. (H and I) The protein expression levels of α-SMA, Col I and Col III were measured by western blot analysis. *n* = 3. *P < 0.05. (J–O) Representative immunofluorescence images of Col III (green), DAPI (blue) and merged images. Scale bar = 20 μm. *n* = 3. (P) RT-qPCR analysis of circHIPK3 expression in normal or hypoxia-treated CFs. *n* = 3. *P < 0.05. *P < 0.05, vs. normal group.

To investigate the effects of hypoxia on fibroblasts, we established a hypoxia model of CFs in vitro, and the CCK-8 assay displayed that cell viability significantly increased after hypoxia for 24 h (Fig. 1A). Next, CFs were subjected to hypoxia for 24 h or left untreated (normal). Our date showed that hypoxia led to significantly increased proliferation and migration of CFs (Figs. 1B–1G) and caused increased expression of markers of myofibroblasts, including α-SMA and collagen types I and III (Col I and Col III) (Figs. 1H and 1I). Accordingly, an immunofluorescence assay revealed that Col III was highly expressed in hypoxia-induced CFs (Figs. 1J–1O). As expected, RT-qPCR demonstrated that circHIPK3 was significantly upregulated in hypoxia-induced CFs (Fig. 1P). These results suggested that hypoxia induced CF proliferation, migration, and phenotypic switching and increased the expression of circHIPK3.

**Figure 2:**
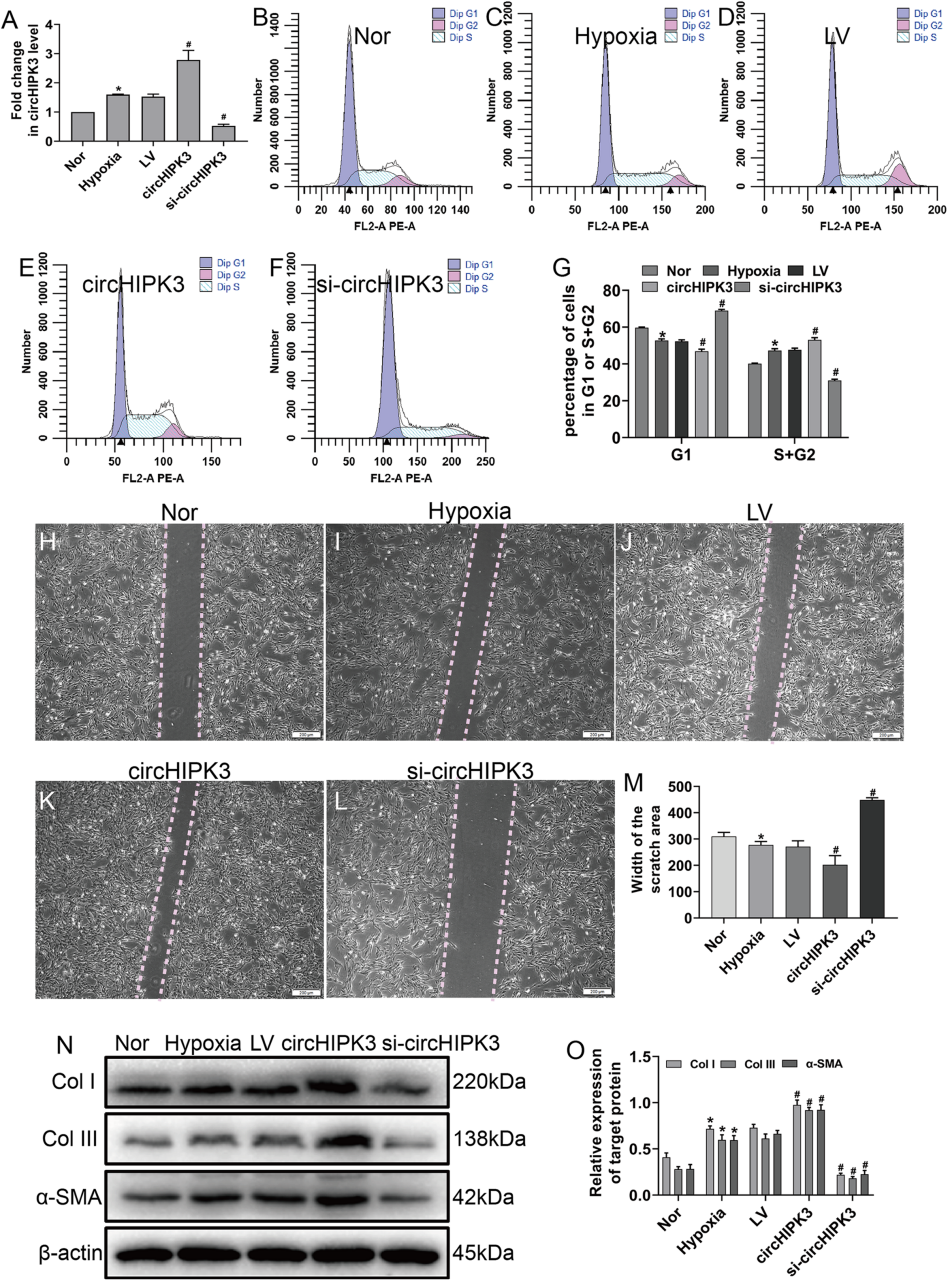
circHIPK3 promoted proliferation, migration and phenotypic modulation in cardiac fibroblasts exposed to hypoxia. CFs were transfected with negative control (LV), ****circHIPK3, or circHIPK3 siRNA (si-circHIPK3) for 48 h. (A) The relative expression of circHIPK3 ****in CFs after different treatments was examined by RT-qPCR analysis. *n* = 3. *P < 0.05, ^#^*P* < 0.05. (B–G) The cell cycle was ****investigated by a cell cycle assay in cardiac fibroblasts. The results represent the percentage ****of cells in G1 and S + G2/M phases for the indicated conditions. *n* = 3. *P < 0.05, ^#^*P* < 0.05. (H–M) Migration was determined ****by the wound scratch assay. The average sizes of the gaps were measured at 48 h. Scale bar ****= 200 μm. *n* = 5. *P < 0.05, ^#^*P* < 0.05. (N and O) The protein expression levels of α-SMA, Col I and Col III were measured by ****western blot analysis. *n* = 3. *P < 0.05, ^#^*P* < 0.05. *P < 0.05, vs. normal group. ^#^*P* < 0.05, vs. hypoxia group.

Hypoxia clearly enhanced circHIPK3 expression in CFs, so we further explored the effect of circHIPK3 in CFs. We performed gain- and loss-of-function analyses of circHIPK3, in which CFs were transfected with circHIPK3 and si-circHIPK3. After 48 h, the transfection efficiency was examined by RT-qPCR. The results showed that circHIPK3 transfection significantly upregulated circHIPK3 expression and that si-circHIPK3 transfection was downregulated its expression (Fig. 2A). CircHIPK3 overexpression significantly promoted proliferation and migration and increased the expression of α-SMA, Col I and Col III. In contrast, circHIPK3 silencing (si-circHIPK3) attenuated proliferation, migration, and α-SMA, Col I and Col III expression (Figs. 2B–2O). Our date confirmed that circHIPK3 can accelerate the phenotypic switch of CFs under hypoxia in vitro.

### CircHIPK3 could bind to miR-152-3p

**Figure 3:**
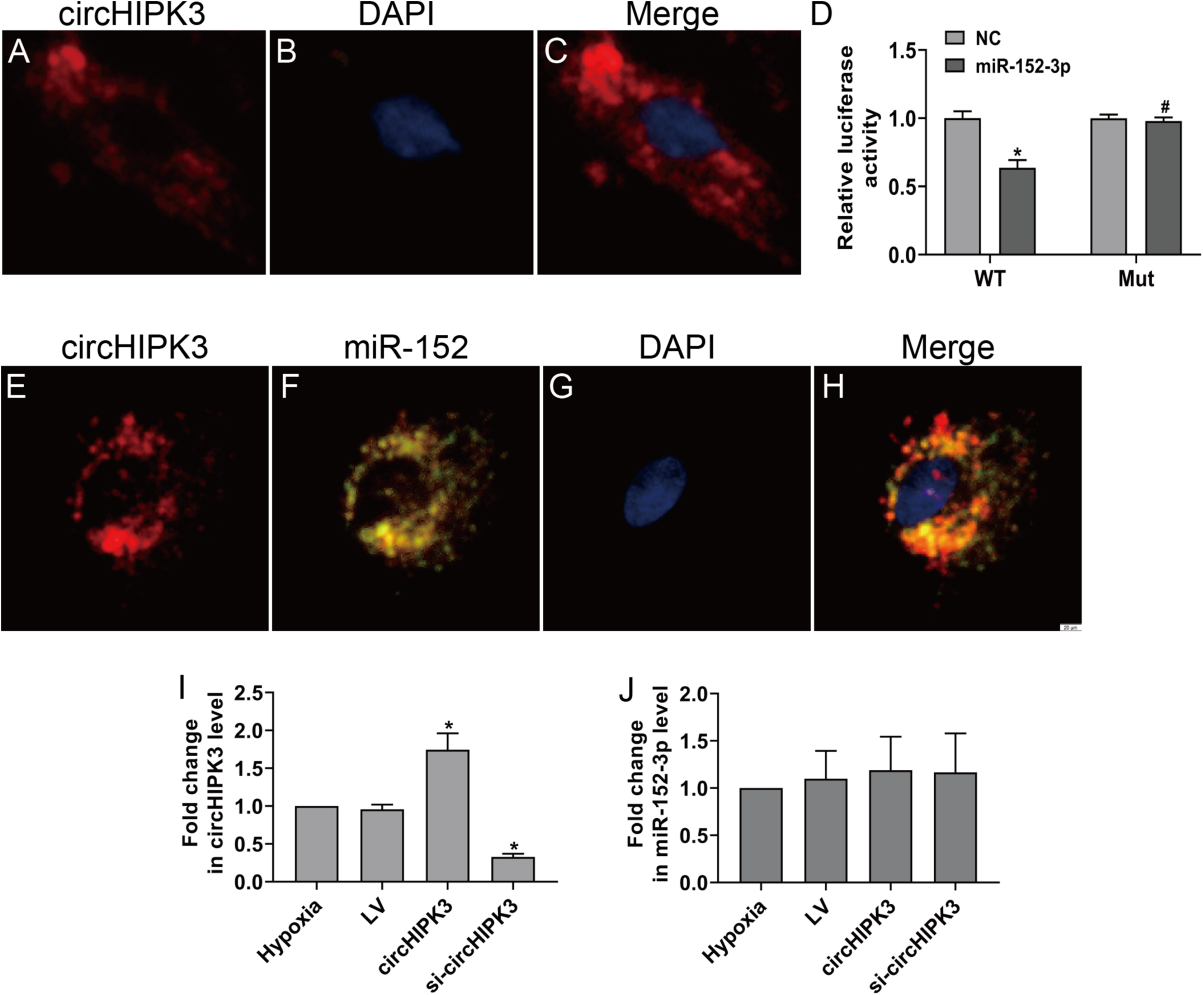
circHIPK3 acts as a miR-152-3p sponge. (A–C and E–H) RNA FISH assays were conducted to detect circHIPK3 and miR-152 localization in CFs using a Cy3-labeled circHIPK3 probe and FITC-labeled miR-152 probe. Nuclei were stained with DAPI. Scale bar = 20 μm. *n* = 3. (D) A luciferase reporter assay was conducted to detect the luciferase activity of RLuc-circHIPK3-WT and RLuc-circHIPK3-Mut in HEK-293T cells transfected with miR-152-3p mimics or NC mimic. Luciferase activity was detected 48 h after transfection. *n* = 3. *P < 0.05, vs. circHIPK3-WT + NC group. ^#^*P* < 0.05, vs. circHIPK3-WT + miR-152-3p group. (I and J) Relative expression of circHIPK3 and miR-152-3p in CFs after different treatments was examined by RT-qPCR analysis. *n* = 3. *P < 0.05, vs. hypoxia group.

CircHIPK3 has been shown to be predominantly existed in the cytoplasm (Wang et al., 2019b). The RNA FISH assay also revealed that circHIPK3 was predominantly located in the cytoplasm of CFs (Figs. 3A–3C). CircRNAs in the cytoplasm are mainly involved in biological processes through sponge-like binding of miRNAs (Li et al., 2015; Xin et al., 2017). To determine whether circHIPK3 could sponge miRNAs, bioinformatics prediction using the StarBase database indicated that miR-152-3p was predicted to bind to circHIPK3. We further carried out a dual luciferase reporter assay in which RLuc-circHIPK3-WT or RLuc-circHIPK3-Mut was cotransfected with miR-152-3p mimic or NC mimic into HEK-293T cells. MiR-152-3p mimic transfection markedly reduced the luciferase activity of RLuc-circHIPK3-WT, but the luciferase activity of the mutant was not affected (Fig. 3D). RNA FISH assays revealed that circHIPK3 and miR-152-3p were colocalized in the cytoplasm of CFs (Figs. 3E–3H). However, circHIPK3 did not affect the expression of miR-152-3p (Figs. 3I and 3J).These results indicated that circHIPK3 could bind to miR-152-3p.

### MiR-152-3p inhibits cell proliferation and phenotypic transformation by targeting TGF-β2

**Figure 4:**
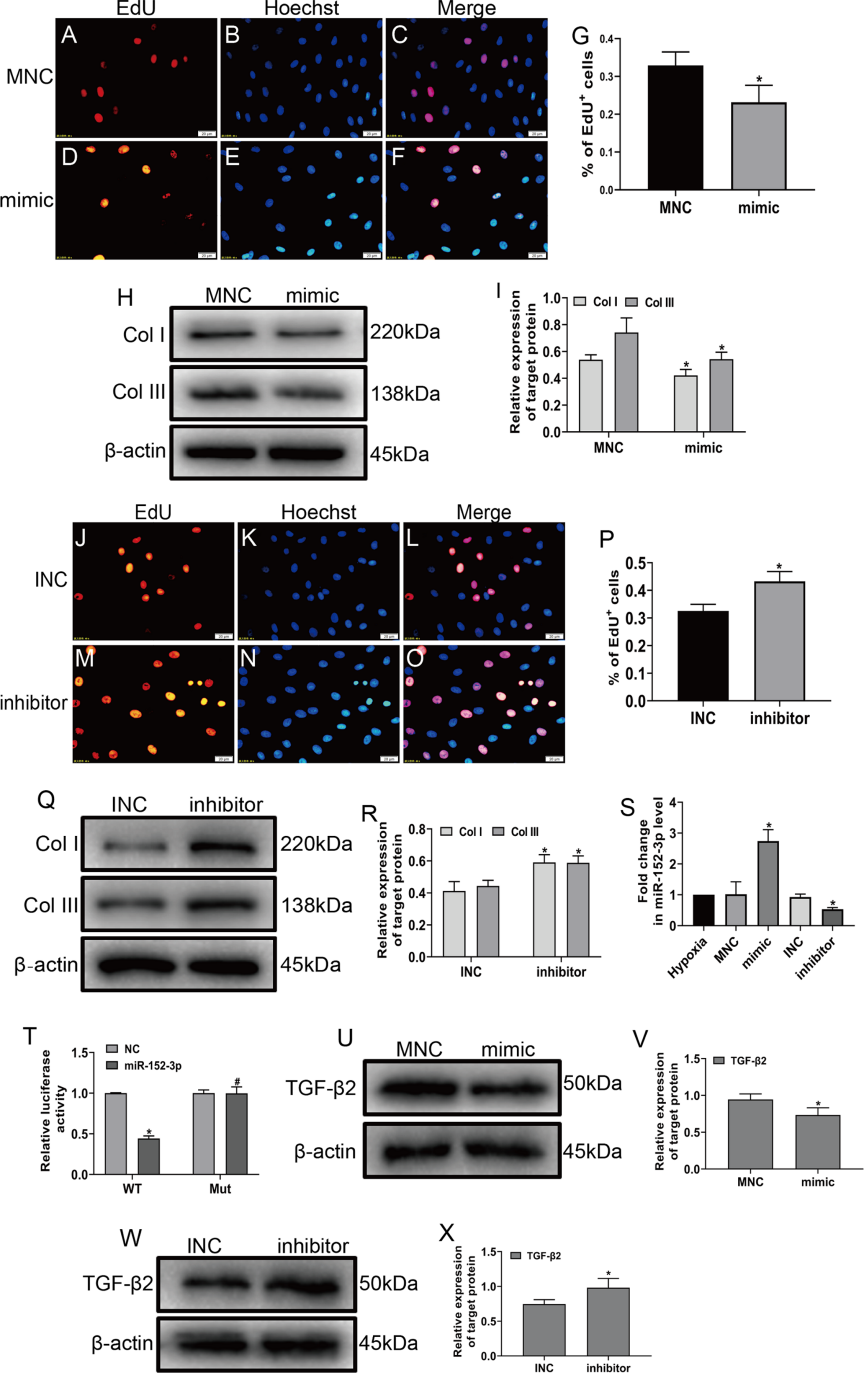
MiR-152-3p inhibited the phenotypic transformation by targeting TGF-β2. To determine the role of miR-152-3p in CFs, CFs were transfected with the miR-152-3p mimic or inhibitor. (A–G and J–P) Representative images of EdU-stained CFs from different groups. Quantification of EdU+ cells presented as the % EdU-positive cells and Hoechst-stained nuclei. The orange color is EdU-positive cells, and the blue color is nuclei stained by Hoechst 33342. Scale bar = 20 μm. *n* = 10. *P < 0.05, vs. MNC group or INC group. (H, I and Q, R) The protein expression levels of Col I and Col III were measured by western blot analysis. *n* = 3. *P < 0.05, vs. MNC group or INC group. (S) Relative expression of miR-152-3p in CFs after transfection with miR-152-3p mimic or inhibitor was examined by RT-qPCR analysis. *n* = 3. *P < 0.05, vs. hypoxia group. (T) TGF-β2 was predicted as a target gene of miR-152-3p using the TargetScan database. HEK-293T cells were cotransfected with RLuc-TGF-β2-WT or RLuc-TGF-β2-Mut and miR-152-3p mimics or NC mimic. Luciferase activity was detected using the dual luciferase reporter assay at 48 h post-transfection. *n* = 3. *P < 0.05, vs. TGF-β2-WT + NC group. ^#^*P* < 0.05, vs. TGF-β2-WT + miR-152-3p group. (U–X) The protein expression level of TGF-β2 was measured by western blot analysis. *n* = 3. *P < 0.05, vs. MNC group or INC group.

To explore the role of miR-152-3p in CFs, we examined its role in CFs under hypoxic conditions. The miR-152-3p mimic, miR-152-3p inhibitor, NC mimic, and NC inhibitor were transfected into CFs. The enforced expression of miR-152-3p in CFs using a mimic inhibited CF proliferation and the expression of Col I and Col III after hypoxia stimulation (Figs. 4A–4I). In contrast, miR-152-3p knockdown in CFs resulted in an augmentation of fibrosis, including cell proliferation and expression of the fibrotic markers Col I and Col III (Figs. 4J–4R). Moreover, we found that the miR-152-3p mimics significantly increased and the miR-152-3p inhibitor significantly decreased miR-152-3p expression in CFs (Fig. 4S).

MicroRNA play a vital biological role by regulating mRNA degradation through complete or incomplete pairing with the 3′ untranslated region of mRNA and then inhibiting gene expression (Boon & Dimmeler, 2015; Hodgkinson et al., 2015; Wang et al., 2018). We screened various fibrosis-associated genes Smad2, Smad3, TGF-β1, and TGF-β2. Then, we searched the TargetScan database to predict miR-152-3p targets and found that miR-152-3p has binding sites on Smad2 and TGF-β2, and we selected TGF-β2 as the target gene for our study based on the context++ score percentile. Ultimately, we identified TGF-β2 as a downstream target of miR-152-3p. Furthermore, TGF-β2 was confirmed as a target of miR-152-3p by the dual luciferase reporter assay (Fig. 4T). In addition, the miR-152-3p mimic markedly reduced the expression of TGF-β2 protein, while downregulation of miR-152-3p clearly increased TGF-β2 protein expression (Figs. 4U–4X). Thus, these results revealed that miR-152-3p participates in antagonizing the phenotypic transformation by targeting TGF-β2.

### CircHIPK3 promotes phenotypic transformation via miR-152-3p/TGF-β2 in cardiac fibroblasts

**Figure 5:**
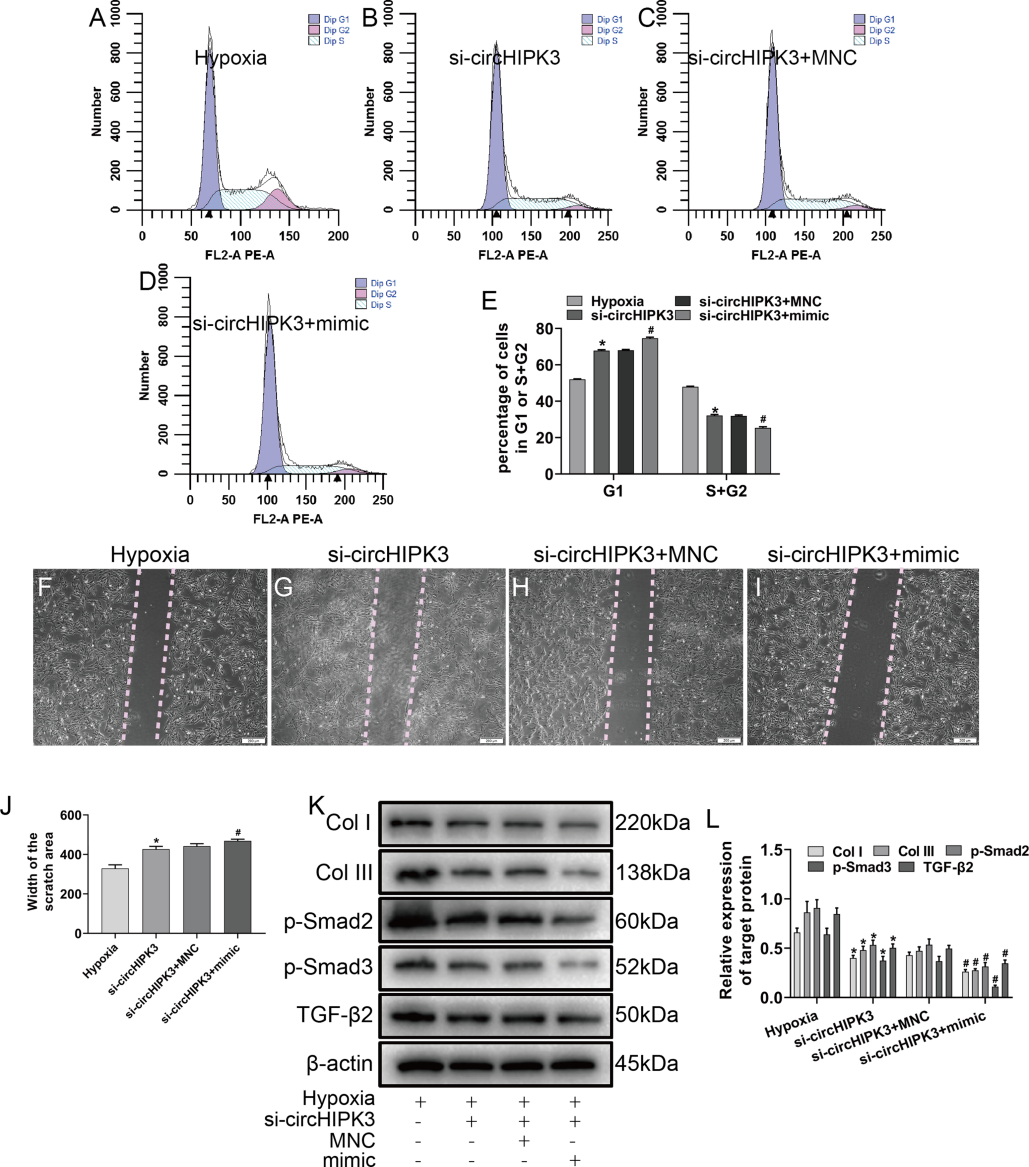
circHIPK3 sponges miR-152-3p and inhibits its activity. (A–E) The cell cycle was investigated by a cell cycle assay in cardiac fibroblasts. The results represent the percentage of cells in G1 and S + G2/M phases for the indicated conditions. *n* = 3. *P < 0.05, ^#^*P* < 0.05. (F–J) Wound scratch assay for the different groups. The average sizes of the gaps were measured at 48 h. Scale bar = 200 μm. *n* = 5. *P < 0.05, ^#^*P* < 0.05. (K and L) The protein expression levels of Col I, Col III, TGF-β2, p-Smad2 and p-Smad3 were measured by western blot analysis. *n* = 3. *P < 0.05, ^#^*P* < 0.05. *P < 0.05, vs. hypoxia group, ^#^*P* < 0.05, vs. si-circHIPK3 group.

To explore whether circHIPK3 was involved in miR-152-3p-mediated regulation of proliferation, migration and fibrosis of CFs in vitro, the cells were cotransfected with circHIPK3 and miR-152-3p. Our data illustrated that circHIPK3 silencing could suppress cell proliferation and migration. Furthermore, the combination of circHIPK3 silencing and miR-152-3p overexpression had a weaker effect under hypoxia than circHIPK3 knockdown alone (Figs. 5A–5J). To evaluate whether circHIPK3 exerted its functions by regulating TGF-β2 expression, we measured the effect of circHIPK3 by western blotting. The results demonstrated that circHIPK3 silencing attenuated the expression of TGF-β2 and the fibrosis markers Col I and Col III. Moreover, it decreased the expression of Smad2 and Smad3, which are downstream proteins of TGF-β2. MiR-152-3p overexpression promoted the inhibitory effect of circHIPK3 silencing in CFs (Figs. 5K and 5L). Collectively, these results indicated that circHIPK3 can accelerate fibrosis progression via the functional pathway of sponging miR-152-3p.

## Discussion

In the present study, we characterized one of the most abundantly expressed and conserved circRNAs, circHIPK3, which is highly associated with cell proliferation, migration, angiogenesis, and fibrosis in the pathogenesis of several cardiovascular diseases (Ni et al., 2019; Shan et al., 2017; Wei et al., 2020; Zhang et al., 2019a; Zheng et al., 2016). Here, we provide compelling evidence that circHIPK3 is involved in regulating cardiac fibroblast function and phenotypic transformation. Mechanistically, circHIPK3 could upregulate TGF-β2 expression by sponging miR-152-3p to regulate cardiac fibroblast-to-myofibroblast transition under hypoxia.

In ischemic heart disease, initial cardiac responses to hypoxia are likely organ protective (Watson et al., 2014). Our previous studies have shown that hypoxia-pretreated cardiomyocytes could secrete protective substances (Wang et al., 2019b). Studies have indicated that hypoxia can induce cardiac fibroblast proliferation and phenotypic transformation (Bao et al., 2020; Kumar et al., 2019; Watson et al., 2014). CFs are commonly regarded as the support cells of the cardiac network and play numerous roles in normal cardiac development and cardiac remodeling in pathological conditions (Chacar et al., 2017). We know that there might be differences in behavior and function of adult mouse CFs and neonatal mouse CFs (Bouzegrhane & Thibault, 2002; Souders, Bowers & Baudino, 2009). A major feature of the adult mammalian heart responds to ischemic injury is formation of persistent fibrotic scar tissue. CFs are critical for the structural integrity of the heart after ischemic injury. However, extracellular matrix deposition and formation of persistent scar tissue eventually leads to heart failure (Mizutani, Wu & Nusse, 2016). Similar to the adult heart, the mammalian neonatal heart responds to cardiac injury by the development of transient fibrotic tissue. After apical resection of the neonatal heart for 3 weeks, regenerated cardiomyocytes replaced fibrosis (Porrello et al., 2011; Porrello et al., 2013). At present, studies on the relationship between hypoxia and neonatal mouse CFs are rare. A better understanding of neonatal heart fibrosis may make it possible to treat permanent scarring of the adult heart, which led us to explore the mechanism between hypoxia and the fibrotic phenotype in neonatal mouse CFs.

Circular RNAs are unusually stable RNA molecules with cell- or tissue-specific expression and their covalently closed loop structure protects them from degradation by RNase (Cai et al., 2019; Qu et al., 2017). Emerging evidence has shown that circRNAs participate in a wide range of biological processes, including transcription, mRNA splicing, and translation (Li, Yang & Chen, 2018). CircHIPK3, a “star” molecule, has been proven to be involved in the regulation of cell growth, proliferation, migration, angiogenesis, and so on (Shan et al., 2017; Wei et al., 2020; Zheng et al., 2016). Many studies have indicated that circHIPK3 is involved in complex regulatory networks and has multifunctional roles in diseases. To date, multiple roles of circHIPK3 in fibrosis have been reported (He & Ou, 2020; Ni et al., 2019; Zhang et al., 2019a). However, the role of circHIPK3 in CFs under hypoxic conditions is still unclear; thus, we focused on revealing the function of circHIPK3 in CFs under hypoxia. In this study, we observed that hypoxia promoted the proliferation, migration and phenotypic transformation of CFs. We also found that circHIPK3 was dramatically upregulated in CFs subjected to hypoxia. Thus, we hypothesized that cardiac fibrosis is caused by high expression of circHIPK3. In this study, the proliferation and migration of CFs were enhanced and the expression levels of α-SMA, Col I and Col III were significantly increased after circHIPK3 overexpression. Conversely, these effects were suppressed when circHIPK3 was silenced. In short, our results manifested that the upregulation of circHIPK3 could facilitate fibrosis of CFs under hypoxia.

Studies have revealed that circRNAs principally bind to miRNAs to act as RNA sponges and increase downstream gene expression by inhibiting miRNA activities, thus contributing to disease progression (Song et al., 2020; Wang et al., 2016; Zhao, Chen & Jiang, 2020). Similarly, for circHIPK3, the most classical mechanism for its functional effect is to act as a sponge for miRNAs, blocking the binding of miRNAs with downstream target genes and regulating the expression of target genes. For example, circHIPK3 acts as an endogenous sponge of miR-30a-3p to inhibit the activity of miR-30a-3p and ultimately promote the proliferation and differentiation of myoblasts (Chen et al., 2019). Wei et al. (2020) reported that circHIPK3 was significantly upregulated in gastric cancer tissues and cell lines and could play a pivotal role in the development of gastric cancer via the miR-107/BDNF axis. Liu et al. (2018) demonstrated that circHIPK3 could regulate the function of human lens epithelial cells in vitro through the miR-193a-3p/CRYAA signaling. One study indicated that circHIPK3 could sponge for multiple miRNAs, examples are miR-124, miR-152, miR-193a and miR-29a (Zheng et al., 2016). We predicted circHIPK3-miRNA interactions using the StarBase database and found that miR-152-3p (mmu_miR_152_3p) had a binding site with circHIPK3 (mmu_circ_0001052), which was confirmed by a luciferase reporter assay. We also found that miR-152-3p mRNA levels were not affected whether circHIPK3 was overexpressed or silenced. Subsequently, we predicted the target genes of miR-152-3p using bioinformatics analysis. A wide range of possible genes—including TGF-β2—are involved in the processes of fibrosis by regulating the phosphorylation of Smad2 and Smad3 (Li, Tang & Chen, 2011; Ma et al., 2014). It is interesting that Smad2 is also a downstream target gene for miR-152-3p; however, we have not explored this research direction. To further demonstrate the effect of miR-152-3p through TGF-β2 on the phenotypic switch under hypoxic conditions, we conducted the following experiments. First, a dual luciferase reporter assay confirmed that miR-152-3p can bind to TGF-β2. Second, we performed miR-152-3p gain- and loss-of-function experiments in vitro and found that miR-152-3p could inhibit cell proliferation and phenotypic switching, as well as reduce TGF-β2 expression. In addition, we found that the effect of silencing circHIPK3 on cell phenotypic transformation was further augmented by miR-152-3p. Paradoxically, a previous study manifested that miR-152-3p dramatically increased cell proliferation and the protein expression levels of Col I and Col III by targeting FOXF1 in keloid fibroblasts (Wang et al., 2019a). This may be due to differences in the environment or upstream or downstream molecules of miR-152-3p, resulting in different effects. In short, our data illustrated that circHIPK3 is a molecular regulator of the process of cardiac fibrosis via miR-152-3p/TGF-β2 signaling. Notably, Hipk3 is downstream of miR-152-3p, and it is worth exploring whether circHIPK3/miR-152-3p and Hipk3 can form a regulatory circuit with a biological function.

## Conclusion

In summary, our study demonstrated that the identified circHIPK3 is upregulated in CFs in vitro in a hypoxic environment. Our results indicated that circHIPK3 silencing can reduce proliferation and migration, and suppress the expression of TGF-β2. CircHIPK3 affected cardiac remodeling and maintenance of integrity by promoting proliferation and migration of CFs while facilitating cardiac fibrosis, in part through miR-152-3p/TGF-β2 signaling mechanisms.

## Additional Information and Declarations

### Data Availability

The following information was supplied regarding data availability: Data are available at Zenodo: Liu Weiwei, & Wang Yan. (2020, July 9). CircHIPK3 regulates cardiac fibroblast proliferation, migration and phenotypic switching through the miR-152-3p/TGF-β2 axis under hypoxia. Zenodo. DOI 10.5281/zenodo.3936255.
